# Life-history and hormonal control of aggression in black redstarts: Blocking testosterone does not decrease territorial aggression, but changes the emphasis of vocal behaviours during simulated territorial intrusions

**DOI:** 10.1186/1742-9994-10-8

**Published:** 2013-02-21

**Authors:** Beate Apfelbeck, Kim G Mortega, Sarah Kiefer, Silke Kipper, Wolfgang Goymann

**Affiliations:** 1 Abteilung für Verhaltensneurobiologie, Max-Planck-Institut für Ornithologie, Eberhard-Gwinner-Str. 6a, Seewiesen, D-82319, Germany; 2 AG Verhaltensbiologie, Institut für Biologie, Freie Universität, Berlin; 3 Abteilung für Ornithologie, Universität Konstanz, Germany

**Keywords:** Androgen receptors, Aromatase, Breeding season, Estradiol, Flutamide, Letrozole, Song, Song structure, Life-history

## Abstract

**Introduction:**

Many studies in behavioural endocrinology attempt to link territorial aggression with testosterone, but the exact relationship between testosterone and territorial behaviour is still unclear and may depend on the ecology of a species. The degree to which testosterone facilitates territorial behaviour is particularly little understood in species that defend territories during breeding and outside the breeding season, when plasma levels of testosterone are low. Here we suggest that species that defend territories in contexts other than reproduction may have lost the direct regulation of territorial behaviour by androgens even during the breeding season. In such species, only those components of breeding territoriality that function simultaneously as sexually selected signals may be under control of sex steroids.

**Results:**

We investigated black redstarts (*Phoenicurus ochruros*), a species that shows periods of territoriality within and outside of the breeding season. We treated territorial males with an anti-androgen and an aromatase inhibitor during the breeding season to block both the direct and indirect effects of testosterone. Three and ten days after the treatment, implanted males were challenged with a simulated territorial intrusion. The treatment did not reduce the overall territorial response, but it changed the emphasis of territoriality: experimental males invested more in behaviours addressed directly towards the intruder, whereas placebo-treated males put most effort into their vocal response, a component of territoriality that may be primarily directed towards their mating partner rather than the male opponent.

**Conclusions:**

In combination with previous findings, these data suggest that overall territoriality may be decoupled from testosterone in male black redstarts. However, high levels of testosterone during breeding may facilitate-context dependent changes in song.

## Introduction

In a reproductive context, testosterone and its metabolite estradiol are considered major hormones facilitating territorial behaviour and the associated vocalizations in a wide range of male vertebrates [e.g. [1,2]. Particularly in birds, seasonal peaks in testosterone closely match periods of intense male-male competition for territories and mates [2]. Furthermore in songbirds, testosterone and estradiol play an important role in the activation of song during the breeding season [reviewed in [3].

Sex steroids, such as testosterone and estradiol, orchestrate physiological, morphological and behavioural changes important for reproduction [e.g. [1]. A close link between the expression of territoriality and testosterone ensures that this behaviour is expressed only in the appropriate breeding life-history context [4], as maintaining high levels of territorial aggression can be energetically costly and may impair survival [5,6]. However, for example in songbirds, it is quite common that males also defend territories outside a breeding context, when testes are regressed and testosterone levels are low (Table 1, [7,8]). When behaviour is expressed over a longer period of time, throughout the year, or in different life-history stages its control may be decoupled from hormones [e.g. 4, see also [9]. Hence, when territorial behaviour occurs in many life-history contexts (i.e. most time of the year) it may be independent of testosterone. Only few species have been studied in this regard: these studies suggest that testosterone plays a role in the regulation of breeding season territoriality also in species that defend territories outside a breeding context (Table 1). However, the degree to which testosterone facilitates territoriality appears to differ between species. The following three scenarios might explain these differences.

**Table 1:** Effects of androgen receptor blocker and/or aromatase inhibition treatment on territoriality and aggression in different species of birds

**Species**	**Treat**	**Days**	**Song output**	**Song str.**	**Calls**	**App. lat.**	**Closest app.**	**Spent close**	**Lat. attack.**	**Flights chases attacks pecks**	**Threat display**	**Loss**	**Reference**
**Breeding season**													
**Songbirds**													
European stonechat	AR/Aro	7-17					**↑**		**↑**			no	[56,57]
European robin	AR	8-14	no			no	no	no				no	[15]
		18-25	no			**↑**	no	no				no	
Song sparrow (pre-breeding)	AR	18	no			no	no	no		**↓**		no	[17]
Song sparrow	AR	18	no			no	no	no		no		no	[17]
Song sparrow	Aro	24hrs	no			no	no	no		no		no	[12]
		8-10	no			no	no	no		no		no	
Red-winged blackbird(*) (polygynous)	AR/Aro	2-5	**no **^**1**^		no					no		**no **^**2**^	[28]
Red-winged blackbird(*)	AR	4-12	no		no					no		no	[28]
Great tit	AR/Aro	2-5	**↓ **^**3**^	no ^4^								no	[32]
Spotted antbird	AR/Aro	8	**↓ **^**5**^		**less snarls**						no	lab	[29]
Rufous-collared sparrow	AR/Aro	7-13	no			no	no	no		no		no	[58]
House sparrow (x)	AR	7-10										no^6^	[59]
**Non-songbirds**													
Corncrake	AR	2			**↓**			**↓**				no	[60]
Bobwhite quail	AR	20								**↓**		lab	[61]
Screech owl	AR/Aro	7-14	no				no	**↓**				no	[62]
Japanese quail (#)	Aro	1-10								**↓**		lab	[45]
Japanese quail (#)	AR	1-10								no		lab	[45]
**Non-breeding season**	**treat**	**days**	**song output**	**song str.**	**calls**	**app. lat.**	**closest app.**	**spent close**	**lat. attack**	**flights, chases**	**threat display**	**loss**	**references**
**Songbirds**													
European stonechat	AR/Aro	7-17					no		no			no	[56]
European robin	AR	6	no			no	no	no				no	[15]
		31-39	no			no	no	no				no	
Song sparrow	Aro	24hrs	no			no	**↑**	no		**↓**		no	[12,63]
		9-12	**↓**			**↑**	**↑**	**↓**		**↓**		no	
Song sparrow	AR/Aro	7	no			no	no	no		no		no	[27]
		30	**↓**			**↑**	**↑**	**↓**		**↓**		no	
Spotted antbird	AR/Aro	8	no		no						no	lab	[51]
Red-winged blackbird(+)	AR	1-15								**↓**		**yes**	[64]
**Non-songbirds**													
Red grouse	AR/Aro	14-21			no							no	[65]
Screech owl (n = 2 – 3)	AR/Aro	7-14	no				no	(**↓**)				no	[62]

“Treat” indicates the type of blocker treatment with either AR (androgen receptors blocked) and/or Aro (aromatase and thus the conversion of androgens to oestrogens blocked). “Song str.” = changes in song structure; “app. lat” = latency to approach a decoy; “closest app.” = closest approach to a decoy; “spent close” = time spent close to a decoy; “lat. attack” = latency to attack a decoy; “loss” = loss of territory or loss of dominance. All species except spotted-antbirds and rufous-collared sparrows were non-tropical species. Most of the studies assessed territorial behaviour by challenging free-living treated territory owners with simulated territorial intrusions, except:

(*) naturally occurring territorial aggression,

(^x^) observation of nest site defence.

(+) lab studies that quantified aggression and dominance between group-housed males.

(#) lab study assessing locomotor activity and pecking rate in response to a stimulus female behind glass.

Numbers in superscript refer to:

^1.^More vocalizations in general.

^2.^Some males lost parts of their territories.

^3.^The likelihood of dawn song was reduced.

^4.^There was no effect on song duration or repertoire size.

^5.^They sang less spontaneous song, less song towards females and during STIs.

^6.^Defence of nest site decreased in AR inhibited individuals, but no loss.

If there was an effect of the treatment, upward arrows indicate that the respective behaviour increased (↑), while downward arrows indicate a decrease in the behaviour (↓). Because of major methodological differences we do not present the study by Archawaranon and Wiley (1988, white-throated sparrows, Aro and testosterone treatment combined) in the table.

First, in some species the intensity of territorial aggression differs between breeding and non-breeding contexts with males expressing only low levels of territorial aggression outside the breeding season. This low-intensity territorial behaviour may be independent of testosterone, but testosterone may intensify territoriality in a breeding context (e.g. mountain spiny lizards, *Sceloporus jarrovi*[10,11], European nuthatches, *Sitta europea*, [9]).

Second, testosterone may facilitate territoriality in breeding and non-breeding contexts, but the source of testosterone may depend on the life-history stage. Song sparrows, *Melospiza melodia*, show similar levels of territorial aggression during the breeding and the non-breeding season [7]. In the non-breeding season, when the circulating testosterone levels of song sparrows are low, testosterone may be produced locally in the brain by conversion of non-gonadal dehydroepiandrosterone (DHEA, [12,13]).

Third, during the breeding season sex steroids may activate exclusively those components of territorial behaviour that are relevant in the breeding context. Territoriality consists of a variety of behaviours including vocalizations (song, calls), spatial behaviours, threat displays and direct aggression. Similar to courtship displays [14] these different components may be facilitated by different (hormonal) pathways [15-17]. During the breeding season, testosterone may specifically activate those aspects of the territorial response that also involve signalling to females. For example, in the grey partridge, *Perdix perdix,* testosterone manipulations affected the quality of the rusty gate call and its salience for females [18,19]. The same pattern may account for the results on territorial behaviour found in most of the bird species studied so far as a strong overall effect of testosterone on territorial behaviour has been the exception rather than the rule (Table 1): birds implanted with androgen receptor blockers (and aromatase inhibitors) did not lose their territories. In most cases the treatment only reduced some aspects of the territorial behaviour or had no effect at all on any of the behaviours measured. In addition, in studies where blocking the action of testosterone had an effect on territorial behaviour, this effect was usually found during the breeding life-history stage, but not outside a breeding context (Table 1).

**Figure 1:**
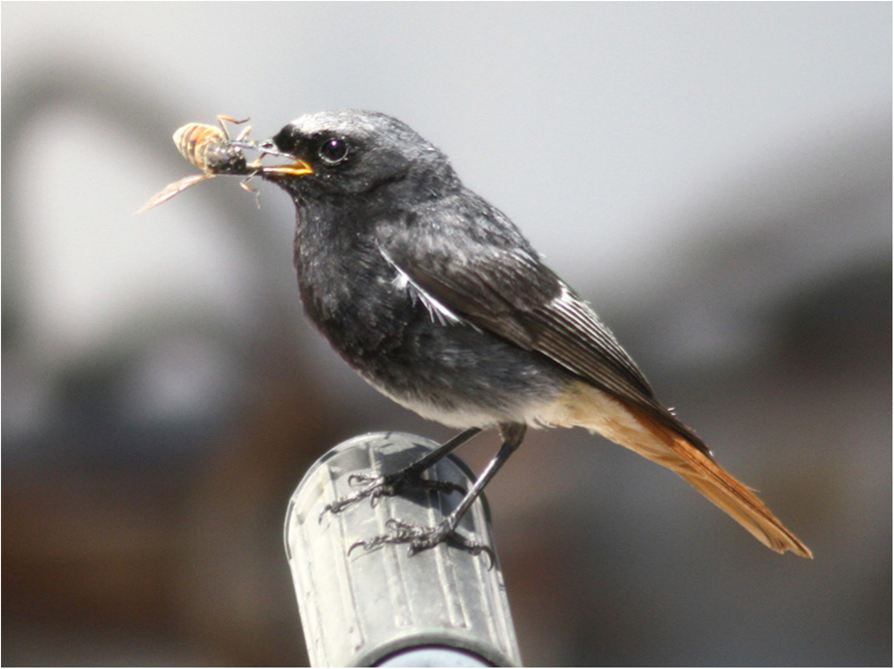
Photograph of an adult male black redstart during breeding.

The aim of this study was to investigate the role of testosterone and its metabolite estradiol in the regulation of breeding season territoriality in short-distance migratory male black redstarts, *Phoenicurus ochruros* (Figure 1)*.* We hypothesize that in this species only some aspects of territoriality may be facilitated by testosterone during breeding, thus supporting the third scenario described above. Males of this species are highly territorial in spring when their testosterone levels are elevated, but also in autumn, just before migration, when testosterone is basal [20]. In both life-history contexts they express androgen and oestrogen receptors and aromatase in brain areas that are relevant for song, sexual and aggressive behaviours [21]. Unlike some other species (reviewed in [22]), male black redstarts do not increase testosterone during agonistic encounters with other males or during simulated territorial intrusions (STI) with a male decoy [20,23].

Accumulating evidence suggests that in this species non-vocal territorial behaviours are independent of testosterone while song output and structure are regulated by testosterone or its metabolites. In black redstarts males responded equally aggressive to a simulated territorial intruder during breeding and non-breeding, but were less likely to sing in response to the intrusion during non-breeding [20]. Furthermore, structural changes in the song in response to simulated territorial intruders seem to depend on testosterone or estradiol in the breeding life-history stage [26]. These song structures are probably indicative of male quality or the male`s ability and/or motivation to defend a territory as they are characteristic of adult males` song compared to song of yearling males [24]. Adult males usually have better territories and a higher breeding success than yearling males [25]. Furthermore, these song structures were enhanced in the agonistic context [26]. Based on these findings we hypothesized that the territorial behaviour as such should be decoupled from the control of sex steroids. Only some components of territoriality (e.g. song structure) that are particularly relevant in a mating and breeding context should be influenced by sex steroids.

We implanted male black redstarts with the anti-androgen flutamide (Flut) and the aromatase inhibitor letrozole (Let) and challenged them with a simulated territorial intrusion (using a mounted decoy and audio-playback of black redstart song). As we were interested in the activational effects of testosterone on territorial behaviour, we tested males already 3 days after implantation. However, as in some studies effects of anti-androgen and aromatase inhibition became only apparent after a longer period of time [15,27], we challenged males a second time 10 days after implantation. We predicted that the ability of Flut/Let-treated males to defend a territory should not differ from that of control males during the breeding season. Further, we predicted that the intensity of non-vocal territorial behaviours should not differ between placebo- and Flut/Let-implanted males. However, based on our previous findings males implanted with Flut/Let should invest less into vocal behaviour than placebo-implanted males, resulting in differences in the song responses between groups.

## Results

### Territory maintenance

All placebo- and Flut/Let-implanted males retained their territories during the period when the Flut/Let treatment was effective (~3 weeks). In fact, most of the males, regardless of treatment, still defended the same territory during autumn, i.e. 6 months after the experiment, before they migrated to their wintering grounds (placebo: 9 out of 10, Flut/Let: 8 out of 10).

### Non-vocal behaviour during the STI

**Figure 2:**
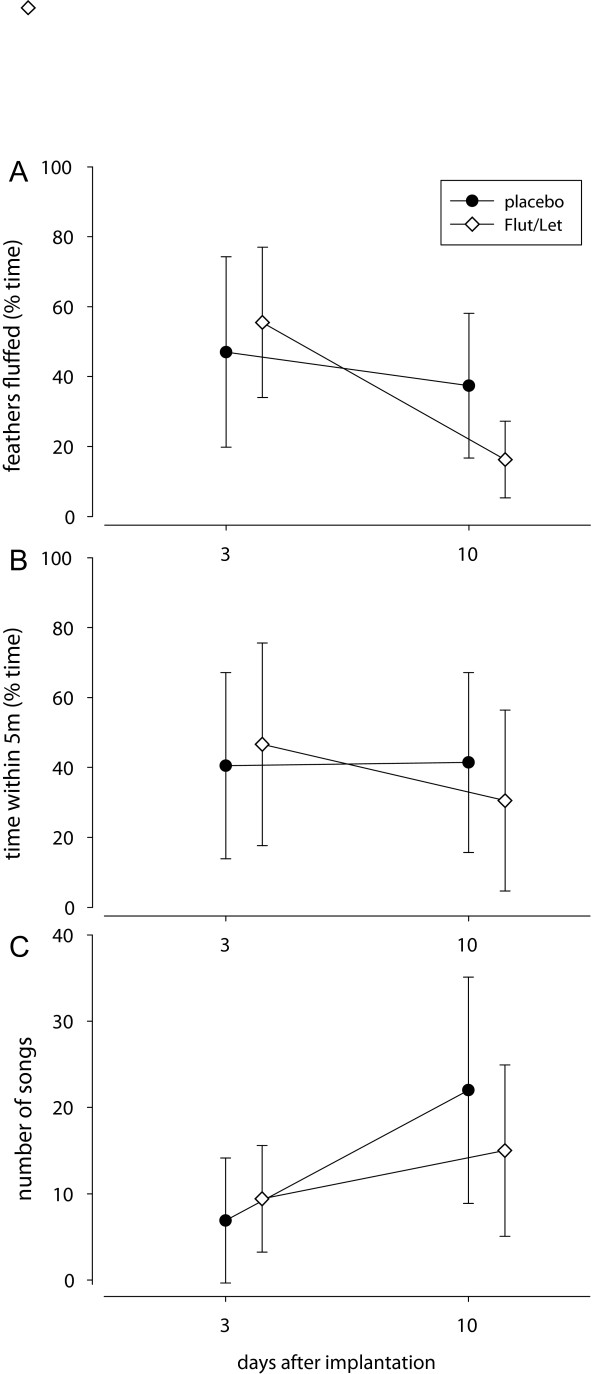
**Non-vocal territorial behaviours (A, B) and number of songs (C).** Behaviours were shown in response to simulated territorial intrusions on day 3 and day 10 after treatment with a placebo or flutamide and letrozole (Flut/Let). Points represent means and error bars represent 95% CI.

Flut/Let and placebo-implanted males did not differ significantly in the time they spent within 5 m of the decoy or in the time they fluffed their feathers. However, for both behaviours day of STI had a significant effect in Flut/Let-, but not in placebo- implanted males: Flut/Let-implanted males spent less time within 5 m of the decoy and less time with their feathers fluffed during the STI on day 10 than during the STI on day 3 (5 m: treatment: F_1,18_ = 0.04, p = 0.8; day: F_1,15_ = 1.6, p = 0.2; interaction: F_1,15_ = 5.1, p = 0.04; feather fluffing: treatment: F_1,18_ = 1.8, p=0.2, day: F_1,15_=12.9, p = 0.003, interaction: F_1,15_ = 7.5, p = 0.02, Figure 2A and B). However, while the two slopes differed significantly, Flut/Let- and placebo implanted males did not significantly differ on day 3 (5 m: t = 0.36, df = 17, p = 0.7; feather fluffing: t = 0.6, df = 16, p = 0.6) and day 10 (5 m: t = - 0.7, df = 18, p = 0.5; feather fluffing: t = - 2.0, df = 14, p = 0.06, Figure 2A and B).

The latency to approach the decoy, the number of head nods and the flights over the decoy did not differ between placebo- and Flut/Let-implanted males (approach latency: treatment: F_1,18_ = 0.15, p = 0.7, day: F_1,16_ = 0.14, p = 0.7; interaction: F_1,16_ = 0.37, p = 0.6; head nods: treatment: F_1,18_ = 0.1, p = 0.7, day: F_1,15_ = 3.0, p = 0.1, interaction: F_1,15_ = 2.0, p = 0.2; flights: treatment: F_1,18_ = 1.6, p = 0.2; day: F_1,16_ = 2.3, p = 0.1; interaction: F_1,16_ = 1.9, p = 0.2). Three males attacked the decoy during the STI on day 3. All of them were implanted with flutamide and letrozole. On day 10 only one of these same males attacked the decoy.

Overall these data suggest that Flut/Let-implanted males showed a reduced non-vocal response on day 10 compared to day 3, because they spent less time close to the decoy and with their feathers fluffed. In placebo-implanted males the non-vocal response did not significantly differ between the STIs on day 3 and 10.

### Vocal behaviour during the STI

**Figure 3:**
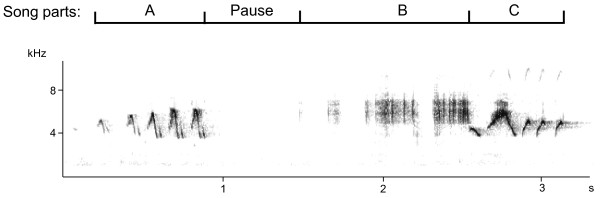
**Spectrogram of one song of a black redstart (Avisoft-SASLab Pro, sample rate 22.05 kHz, FFT = 256 points, hamming-window, overlap: 50%).** Song parts are indicated on top of the spectrogram. Measures analysed were durations of parts **A, B, C,** of the total song and the pause duration between **A** and **B**; the number of elements of part **A** and **C**; the frequency bandwidth and the maximum frequency of part **A, B, C** (see text and [26] for further details).

**Figure 4:**
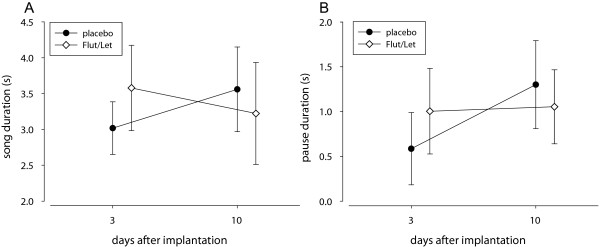
**The duration of songs (A) and of pauses between part A and B (B).** Behaviours were shown in response to simulated territorial intrusions on day 3 and day 10 after treatment with a placebo or flutamide and letrozole (Flut/Let). Placebo-implanted males sang significantly longer songs with a longer pause between part A and B on day 10 than on day 3. Bars represent means and 95% CI.

**Figure 5:**
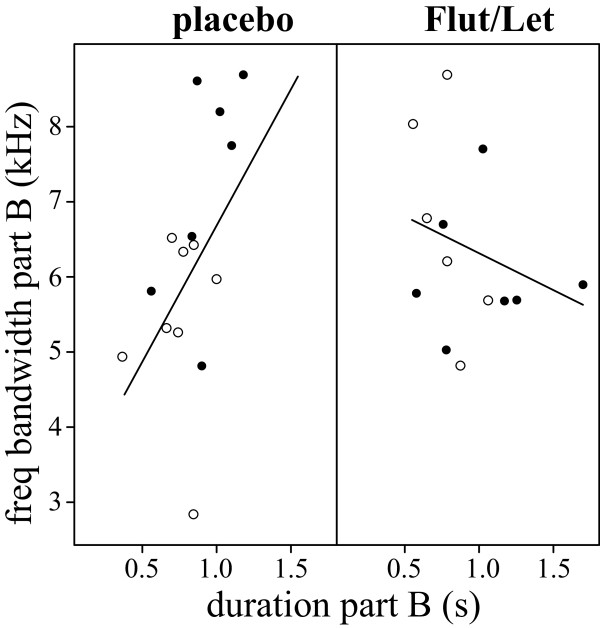
**The frequency bandwidth (kHz) of part B plotted against the duration of part B of songs sang in response to the STI.** Data are shown separately for placebo- (left) and Flut/Let-treated males (right;filled circles: STI on day 3; open circles: STI on day 10). Lines represent regression lines for both days taken together.

There was no direct effect of the treatment on song output: placebo- and Flut/Let-implanted males did not differ in the number of songs they sang in response to the STI. However, males of both groups sang significantly more songs during the STI on day 10 than on day 3 (treatment: F_1,18_ = 0.04, p = 0.8; day: F_1,16_ = 13.4, p = 0.002, interaction: F_1,16_ = 0.4, p = 0.5; Figure 2C). In measures of song structure (see Figure 3 for a typical black redstart song), we also could not find any direct effect of treatment, but in several measures the treatment interacted with day of STI. Placebo-implanted males sang significantly longer songs on day 10 than on day 3 (Table 2, Figure 4A). This was mainly due to a longer pause duration between part A and B on day 10 than on day 3 (Table 2, Figure 4B). There was no clear change in Flut/Let- implanted males for both of these measures (Table 2, Figure 4). Placebo-implanted males sang part B with a significantly broader frequency bandwidth and with a longer duration on day 3 than on day 10, while there was no clear change in Flut/Let-implanted males (Table 2, Figure 5). Furthermore, in placebo-implanted males the relationship between frequency bandwidth and duration of part B was positive, i.e. males that sang parts B with a long duration also sang them with a broad frequency bandwidth (Figure 5). In contrast, this relationship was negative in Flut/Let-implanted males, i.e. birds that sang parts B with larger bandwidths sang these shorter (Figure 5). When controlling for the duration of part A, males of both treatment groups tended to sing it with fewer elements on day 10 than on day 3 (Table 2). Thus, both treatment groups sang more songs, but with supposedly lower competitive value during the STI at day 10 than at day 3 as they sang song that was more similar to the song of yearling than to the song of adult males ([24], lower frequency bandwidth and duration of part B in placebo-implanted males, longer songs with longer pause durations between part A and B, less elements in part A). Surprisingly though, the decrease in competitive value of the song from day 3 to day 10 was much more pronounced in placebo- than in Flut/Let-implanted males.

**Table 2:** Linear mixed model results for the effects of treatment (placebo- or Flut/Let-implanted) and day (3 or 10) after implantation on vocal behaviours

	**During STI**		**After STI**	
**Song duration**				
Treatment	F_**1,16**_ = 0.07	p = 0.8	F_1,18_ = 2.8	p = 0.1
Day of STI	F_**1,12**_ = 0.2	p = 0.7	F_1,17_ = 0.3	p = 0.6
Treatment*day	**F**_**1,12 **_**= 8.4**	**p = 0.01**		
**Duration pause A - B**				
Treatment	F_**1,16**_ = 0.4	p = 0.6	F_1,18_ = 1.5	p = 0.2
Day of STI	**F**_**1,13 **_**= 5.2**	**p = 0.04**	**F**_**1,13 **_**= 5.8**	**p = 0.03**
Treatment*day	F_1,13_ = 3.8	p = 0.07		
**Elements in A**				
Duration A	**F**_1,12_**=17.8**	**p = 0.001**	F_1,12_ = 0.2	p = 0.7
Treatment	F_1,16_ = 0.1	p = 0.8	F_1,18_ = 0.2	p = 0.7
Day of STI	F_1,12_ = 4.2	p = 0.06	**F**_1,12_**= 6.7**	**p = 0.02**
Duration A*treatment	F_1,12_ = 1.6	p = 0.2	F_1,12_ = 4.0	p = 0.07
Duration A*day			F_1,12_= 0.4	p = 0.5
Treatment*day			F_1,12_ = 0.4	p = 0.5
Duration A*treatment*day			**F**_1,12_**= 4.7**	**p = 0.05**
**Frequency bandwidth A**				
Duration A	F_1,12_ = 2.5	p = 0.1	F _1,16_ = 0.09	p = 0.8
Treatment	F_1,16_ = 1.0	p = 0.3	**F**_1,18_**= 5.2**	**p = 0.04**
Day of STI	F_1,12_ = 0.004	p = 1.0	**F**_1,16_**= 4.4**	**p = 0.05**
Duration A*treatment	F_1,12_ = 3.4	p = 0.09		
**Frequency bandwidth B**				
Duration B	F_1,6_ = 0.07	p = 0.8	F_**1,15**_ = 3.1	0.1
Treatment	F_1,15_ = 0.02	p = 0.9	**F**_**1,18 **_**= 6.2**	**p = 0.02**
Day of STI	**F**_**1,6 **_**= 13.7**	**p = 0.01**	F_**1,15**_ = 4.2	p = 0.06
Duration B*treatment	**F**_**1,6 **_**= 56.1**	**p = 0.0003**		
Duration B*day	**F**_**1,6 **_**= 23.5**	**p = 0.003**		
Treatment*day	**F**_**1,6 **_**= 31.3**	**p = 0.001**		
Duration B*treatment*day	F_**1,6**_ = 4.6	p = 0.07		
**Elements in C**				
Duration C	**F**_**1,11 **_**= 13.7**	**p = 0.004**	**F**_**1,15 **_**= 8.1**	**0.01**
Treatment	F_**1,16**_ = 0.3	p = 0.6	F_**1,18**_ = 0.005	p = 1.0
Day of STI	F_**1,11**_ = 1.2	p = 0.3	**F**_**1,15 **_**= 6.3**	**p = 0.02**
**Frequency bandwidth C**				
Duration C	F_**1,11**_ = 1.3	p = 0.3	F_**1,15**_ = 0.3	p = 0.6
Treatment	F_**1,16**_ = 0.5	p = 0.5	F_**1,18**_ = 0.07	p = 0.8
Day of STI	F_**1,11**_ = 0.01	p = 1.0	F_**1,15**_ = 0.8	p = 0.4

To control for repeated measures the ID of each territory owner was included as random intercept. Significant results are highlighted in bold.

### Non-vocal behaviour after the STI

After the decoy was removed and the playback stopped, placebo- and Flut/Let-implanted males did not differ in the amount of time they spent within 10 m of the decoy (treatment: F_1,18_ = 0.5; day: F_1,18_ = 1.7, p = 0.2; interaction: F_1,18_ = 0.2, p = 0.7), in the number of head nods (treatment: F_1,18_ = 0.2, p = 0.7; day: F_1,17_ = 0.1, p = 0.7; interaction: F_1,17_ = 0.1, p = 0.8) or in the number of songs (treatment: F_1,18_ =0.004, p = 1.0; day: F_1,18_ = 3.1, p = 0.1; interaction: F_1,18_ = 0.4, p = 0.5).

### Vocal behaviour after the STI

**Figure 6:**
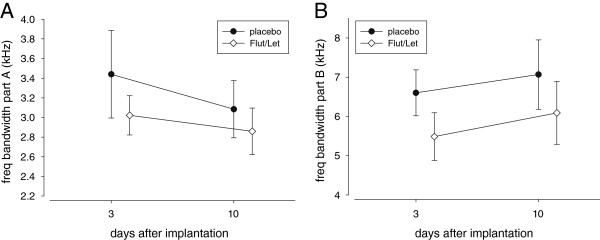
**Frequency bandwidth (kHz) of song parts A (A) and B (B).** The song was recorded after simulated territorial intrusions on day 3 and day 10 after treatment with a placebo or flutamide and letrozole (Flut/Let). Placebo-implanted males sang both parts with a significantly broader frequency bandwidth than Flut/Let-implanted males. For details on song measurements and statistics see text.

After the STI, placebo- and Flut/Let-implanted males differed significantly in song structure: Flut/Let-implanted males sang parts A and B after both STIs with a smaller frequency bandwidth than placebo-implanted males (Table 2, Figure 6).

**Figure 7:**
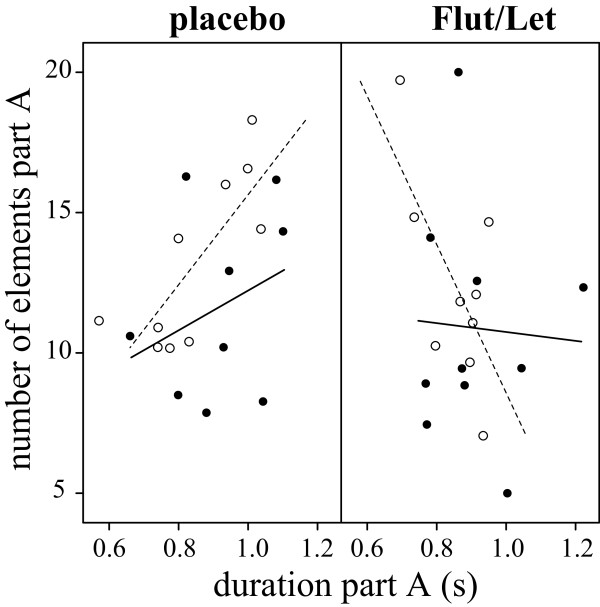
**The number of elements in part A plotted against the duration of the part A of songs sang after the STI.** Data are presented separately for placebo- (left) and Flut/Let-treated males (right; filled circles and solid lines: STI on day 3; open circles and dashed lines: STI on day 10). Lines represent regression lines.

Furthermore, males of both treatment groups changed structural features of their song from day 3 to day 10: they sang with longer pauses between parts A and B on day 10 compared to day 3 (Table 2) and part C with more elements on day 10 than on day 3 (Table 2). In addition, when controlling for the duration of part A, placebo-implanted males sang more elements in this part on day 10 than on day 3 (Table 2, Figure 7). Also, Flut/Let-implanted males tended to repeat more elements in part A on day 10 than on day 3, but they did so with a shorter duration of part A compared to control males (Table 2, Figure 7).

Thus, song sung by Flut/Let-implanted males after the STI was in general of potentially lower competitive value than that of placebo-implanted males (smaller frequency bandwidth of part A and B). In addition, after the STI males of both treatment groups tended to sing song that was probably of higher competitive value on day 10 than on day 3 (more elements in part A and C).

## Discussion

In a breeding context inhibiting the effects of testosterone and oestrogen did not prevent male black redstarts from successfully defending their territories and had no direct effect on the non-vocal territorial response or song output. However, after the STI the song structure of Flut/Let-implanted males differed significantly from that of placebo-implanted males, especially in those parts of their song that males enhance in response to a territorial intruder [26]. Furthermore, we found a surprising effect of day of STI. We had challenged males 3 and 10 days after implantation, because in some studies the effect of pharmacologically inhibiting the action of androgens was only apparent after several days to weeks [15,27]. In line with these studies, the non-vocal territorial response of Flut/Let-implanted males was reduced during the second STI 10 days after implantation compared to the STI only 3 days after implantation. However, when compared directly, the non-vocal territorial response did not differ between placebo- and Flut/Let-treated males, neither on day 3 nor on day 10. Furthermore, in contrast to the expectation that day of STI may have had an effect in Flut/Let-implanted males, it also had an effect in placebo-implanted males: placebo- and Flut/Let-implanted males reduced their vocal response to the STI from day 3 to day 10, but the effect was stronger in placebo- compared to Flut/Let-implanted males. After the respective STIs, males of both groups enhanced their vocal response from day 3 to day 10 (but Flut/Let males less so than placebo-implanted males). Thus, in both treatment groups there was a significant effect of day of STI, which may reflect an effect of experience. We have shown previously in black redstarts, that challenging males with a STI on three consecutive days significantly changed their behaviour towards a territorial intruder: they approached the intruder faster and tended to spent more time close to the decoy on day 3 than on day 1 [23]. The present experiment suggests that there is still an effect of experience even with a gap of 7 days between the experiments and that the treatment significantly influenced this effect. The data suggest that both treatment groups changed the emphasis of the territorial response from during to after the STI from day 3 to day 10. Furthermore, blocking androgen and oestrogen action changed the emphasis of the territorial response during day 3. Placebo-implanted males showed a stronger vocal response (as evident in song structural changes from day 3 to day 10), while experimental males responded more with direct approach and non-vocal threat behaviours (as evident in changes in these behaviours from day 3 to day 10; summarized in Table 3).

**Table 3:** Relative change in behaviour from day 3 to day 10 in response to the STI

		**Placebo**	**Flut/Let**
**Non-vocal response**	Time within 5 m		**↓**
	Feather fluffing		**↓**
**Vocal response**	Number of songs	**↑**	**↑**
	Song duration	**↑**	
	Pause A - B	**↑**	
	Freq bandwidth B (duration part B)	**↓**	
	Elements in A	**↓**	**↓**

Downward arrows (↓) indicate a quantitative decrease of the behaviour from day 3 to day 10; upward arrows (↑) indicate a quantitative increase of the behaviour from day 3 to day 10. The emerging pattern suggests that both treatment groups reduced their territorial response during the STI from day 3 to day 10, but that this change mainly concerned non-vocal behaviours in blocker-implanted males, but vocal behaviours in placebo-implanted males.

Thus, black redstarts reacted similarly to most other songbird species, in which treatment with androgen inhibitors during the breeding life history stage did not reduce overall territorial behaviour, but only some components of it (Table 1, but see [27]) and supports the view that testosterone emphasizes vocalizations (structure and/or output) within the territorial response. For example, male red-winged blackbirds, *Agelaius phoeniceus,* implanted with an androgen receptor blocker and an aromatase inhibitor spent more time on their territories engaging in aggressive interactions and vocalizations compared to control males, but still lost parts of their territories (Table 1; [28]). It is unknown why male red-winged blackbirds implanted with anti-androgens and an aromatase inhibitor were less able to defend their territories, but possibly the treatment could have had an effect on song structure and thereby the quality of the song. This remains speculative, however, because song structure was not measured in the study on red-winged blackbirds. Male spotted antbirds*, Hylophylax n. naevioides*, implanted with anti-androgens and aromatase inhibitors do not sing at all and produce fewer aggressive calls in response to a staged male-male encounter in captivity compared to control males while non-vocal behaviours were not influenced by the treatment [29]. Male European robins, *Erithacus rubecula,* approached intruders more conspicuously by singing from perches above the intruder during the breeding season than during the acquisition of non-breeding territories, even though the quantitative response (latency to approach, time spent close to the intruder, song output) did not differ between seasons [15]. Also in this study song structure was not assessed, but the authors suggested that sexually selected components of the song may be under androgenic influence. Our results strengthen this idea: in species in which territorial behaviour is not restricted to the breeding life history stage, testosterone and estradiol may facilitate only specific components of territorial behaviour that are important in a reproductive, i.e. mating and breeding, context. The specific components that are altered by steroids may, for example, act as signals for females. Song during territorial contests in the breeding season is not only directed towards intruding or neighbouring males, but may also convey information to the mate or other females [30]. Females pay attention to the performance of their mates during territorial challenges, which may influence female behaviour, i.e. the decision whether they engage in extra-pair copulations or not [30]. These features may differ between species (e.g. song rate or song structure), may signal male quality and can be correlated with variation in testosterone levels [31]. In other studies on black redstarts we have shown that males are less likely to respond to a simulated territorial intrusion with song during non-breeding territoriality in autumn (when testosterone levels are low) than during breeding territoriality in spring, while the non-vocal territorial response was not reduced during non-breeding compared to breeding [20]. Furthermore, in male black redstarts the structural changes in song in response to territorial intrusions seem to depend partly on testosterone and/or estradiol, because both males that were implanted with an anti-androgen and an aromatase inhibitor in a breeding context and males that were challenged in a non-breeding context when testosterone levels were naturally low, did not show the full structural change of their song [26]. So far, we are only aware of one further study that incorporated measures of song structure when testing the role of androgens and oestrogens in the regulation of territoriality (see Table 1). This study on great tits, *Parus major*, did not find an effect of anti-androgens and aromatase inhibition on song structure [32]. However, the great tit study assessed effects of the treatment on spontaneously produced dawn song and not song in response to a simulated territorial intruder as in our study. Nevertheless, our data suggest that in male black redstarts and potentially other species as well testosterone and/or oestrogens may shift the focus of the territorial response to vocal behaviours and facilitate structural changes in the song within an agonistic context during the breeding life history stage.

*Dependence of territoriality on androgens and androgen responsiveness to male-male interactions.-* Some bird species show an increase in testosterone after male-male interactions during breeding, whereas others do not (reviewed in [22,33]). These short-term increases of testosterone are thought to enhance the persistence of the territorial response [7], may induce the winner effect [34,35] and are a phenomenon found across all vertebrate classes [2,36-38]. Surprisingly, though, these surges in testosterone are absent in many bird species [22,33]. When territorial behaviour as such is decoupled from the control of testosterone in a reproductive context, as for example in the black redstart, territorial disputes may also not induce short-term increases in testosterone. We have previously shown that male black redstarts do not increase testosterone during simulated and real territorial encounters with other males [20], but obviously this does not prevent them from enhancing their territorial response during future territorial encounters [23]. Song sparrows, in contrast, increase testosterone during simulated territorial intrusions [39] and this increase seems to enhance the persistence of the territorial response after the stimulus is withdrawn [40]. Furthermore, inhibition of aromatase reduces the whole suite of territorial behaviours in response to an intruder in song sparrows, even though the effect is less obvious during the breeding than during the non-breeding season [12]. Why species differ in the hormonal control of aggression displayed in different life history contexts and short-term territorial aggression is still unclear [20,22,33,41,42] and highlights a potential diversity of physiological mechanisms that is largely unexplored [43].

## Conclusions

In some species that defend a territory during and outside a breeding life-history context, territorial behaviour even during breeding may be decoupled from testosterone or its metabolites. Rather, testosterone or estradiol may change particular components of the territorial repertoire that are specifically relevant in a breeding context. Testosterone may shift the emphasis of the territorial response to these components. Thereby, these behaviours may change in signal value and may in turn indicate male quality to other males (intruders and neighbours), but also to females witnessing the territorial dispute. Such context-dependent changes in song structure during the breeding life history stage may be facilitated by seasonal changes in testosterone levels. There seem to be fundamental differences between species to which degree territorial behaviour is regulated by testosterone or its metabolites in different life history contexts. These differences may be directly related to variation in androgen responsiveness to male-male interactions, which in turn may depend on life-history and ecological characteristics of a particular species.

## Material and methods

### Capture and implantation with androgen receptor blockers and aromatase inhibitors

Adult (≥ 2 years) male black redstarts were caught in 2009 between April 9^th^ and 27^th^ in Upper Bavaria (N 47º, E 11º, 500–600 m above sea level) with mealworm-baited ground traps. Birds were lured to the traps by broadcasting playbacks with the species’ song of short duration (< 2 min). We remotely muted the loudspeaker as soon as the territory owner approached the traps. Conspecific playback does not influence testosterone levels in territorial male black redstarts [20,23]. Upon capture we measured the birds and implanted males with either one placebo pellet (n = 10) or two time release pellets (n = 10) containing the androgen receptor blocker flutamide and the aromatase inhibitor letrozole, respectively (21 day release: 1.5 mg per pellet; release rate 71 μg/day; Ø = 3.2mm, Innovative Research of America, Sarasota, FL). Letrozole inhibits cytochrome p450 aromatase (CYP 19). This enzyme is important for the conversion of testosterone to oestrogen [44]. Thus, by combining flutamide and letrozole, it is possible to block direct and indirect effects of androgens on behaviour [45,46]. Implants were inserted subcutaneously with a pair of tweezers through a small incision in the skin on the back between the wings. The incision was sealed with tissue glue (Nexaband; World Precision Instruments).

Control and experimental groups did not differ significantly in body mass (t = 1.52, df = 17.9, p = 0.15), length of the right tarsus (t = -0.25, df = 12.5, p = 0.8), length of the right wing (t = 0.25, df = 14.2, p = 0.8) and cloacal protuberance (CP) volume (t = -0.17, df = 13.1, p = 0.9), which was estimated by calculating the volume of a cylinder (V = π*(CP width/2)^2^*CP height). Each male was banded with a numbered aluminium ring (Vogelwarte Radolfzell) and a unique combination of three colour rings for individual recognition. Measuring, ringing and implanting the birds took no longer than 25 min after which the males were released onto their territories. All experimental procedures were approved by the Committee on the Ethics of Animal Experiments of the governmental authorities of Upper Bavaria (Permit Number: Az. 55.2-1-54-2531-151-08).

### Effectiveness of androgen receptor blockers and aromatase inhibitors

To assess if the treatment with the androgen receptor blocker and the aromatase inhibitor was effective we caught another set of males with the same method as described above in April 2009 and 2010 and in September 2010, took a blood sample immediately upon capture (within 5 min) and brought them to the laboratory. In 2009 five males were caught, implanted with flutamide and letrozole and bled a second time 3 days after implantation. In 2010 we caught another 16 males (8 in April and 8 in September) that were bled upon capture, were then either implanted with flutamide and letrozole or with flutamide only, and bled again 3 and 10 days after implantation. In all cases pellets were still visible when we took the blood samples. Males were held in individual cages under simulated natural photoperiod and released onto their respective territories after taking the last blood sample. Testosterone concentration was determined by direct radioimmunoassay following the procedure described in [20,47]. Mean ± SD efficiency of the extraction with dichloromethane was 93 ± 3% for the samples collected in 2009 and 85 ± 5% for those collected in 2010. Samples were measured in duplicates and in separate assays (2009 + 2010). The lower limit of detection of the assay was determined as the first value outside the 95% confidence intervals for the zero standard (B_max_) and was 2.6 (2009) and 4.5 (2010) pg/ml. The intra-assay coefficients of variation were 1.2% (2009) and 2.9% (2010), respectively. The inter-assay variation was 3.2%.

The combined implantation of flutamide and letrozole significantly increased plasma testosterone levels within 3 days as compared to levels before implantation (paired t-test, n = 9, t = -3.4, p = 0.01, Table 4) and testosterone levels were still significantly elevated after 10 days (Wilcoxon test, U = 0, p = 0.003, Table 4). These data suggest that letrozole effectively inhibited the aromatization of testosterone to oestrogen, because in birds the negative feedback regulation of testosterone is achieved via estradiol which inhibits gonadotropin secretion [27,48,49]. Thus, the increase in testosterone indicates that there is no estradiol that would be capable to induce this negative feedback. As soon as estradiol levels would rise, negative feedback would kick in and as a consequence reduce circulating testosterone levels. This happens when flutamide is implanted without letrozole during the breeding season (Table 4). Hence, elevated testosterone levels provide evidence that letrozole blockage works effectively during breeding (see also [14,27,50-52]). During non-breeding, when the testes are regressed, flutamide and letrozole treatment does not result in an increase in testosterone (Friedman chi-squared = 0.25, df = 2, p = 0.9, Table 4).

**Table 4:** Plasma testosterone levels (ng/ml ± 95% CI) before implantation, and 3 and 10 days after implantation of either flutamide alone or flutamide and letrozole combined

**Testosterone (ng/ml)**	**Before**	**3 Days after**	**10 Days after**
Breeding			
Flutamide/letrozole	1.02 ± 0.4 (n= 9)	5.02 ± 2.0 (n= 9)	5.9 ± 2.1 (n= 9)
Flutamide	2.2 ± 6.6 (n= 4)	2.2 ± 3.4 (n= 4)	1.2 ± 1.5 (n= 4)
Non-breeding			
Flutamide/letrozole	0.06 (n= 4)	0.04 (n= 4)	0.04 (n= 4)
Flutamide	0.04 (n= 4)	0.05 (n= 4)	0.08 (n= 4)

As flutamide is a competitive inhibitor of androgen receptors [53] the increase in testosterone levels caused by letrozole could theoretically have compromised the effectiveness of flutamide. However, this is very unlikely, as the dosage used (1.5 mg flutamide and a release rate of 71 μg/day) resulted in a concentration of flutamide that is roughly 700 times higher than the maximum concentration of testosterone measured in a black redstart. This surplus of flutamide should have been sufficient to effectively inhibit androgen action.

### Playback stimuli

Songs used as playbacks were recorded in spring 2009 with a Sennheiser directional microphone (ME66/K6) connected to a Marantz solid state recorder PMD 660 (sampling frequency: 44.1 kHz; resolution: 16 bit) from 20 different males that were at least 10 km away from our focal males. Playbacks were created using Avisoft Saslab pro software version 4.51 (Raimund Specht, Berlin, Germany, [for details see [26]). Each playback consisted of 20 songs recorded from one male. Each playback was used only once in each experimental group, but the same playbacks were randomly used during the STIs on day 3 and 10. Thus, each male was challenged with two different playbacks.

### Simulated territorial intrusion

To assess the effect of the Flut/Let treatment on territorial behaviour we performed STI experiments three and ten days after implantation by placing a stuffed decoy into the centre of the territory of a focal male and playing back black redstart song as described above. As decoys we used three different stuffed males in full adult plumage that were protected by an inconspicuous cage made of a wire frame and mist net material and mounted on a tripod. A string attached to the wire frame allowed us to remotely remove the decoy by pulling the string from a distance of about 30 m into a plastic cylinder below the wire frame. We put a remote-controlled loudspeaker (Foxpro Scorpion, digital game caller, FOXPRO Inc. Lewistown, USA) underneath the decoy to play back the territorial song of a potential rival at a sound pressure level of 65 dB SPL at 1 m (as measured with a CEL 573.B1 Sound Level Analyser). The behavioural response of male black redstarts to simulated territorial intrusions varies from moving to an exposed singing post and increasing the song output to approaching the decoy and threat posturing, which – in some cases – may cumulate into an attack [16,54]. Therefore, we recorded the following behaviours of the territory owner during the STI for 20 min: (1) latency to respond to the STI either by singing or approaching the decoy, (2) the first time the male entered the area of 5 m around the decoy, (3) the time the male spent within 5 m of the decoy, (4) the time the territory owner spent with its feathers fluffed, (5) the number of head nods and (6) the number of flights over the decoy. The latter two behaviours are typical threat postures of male black redstarts [54]. Furthermore, we noted whenever the male attacked the decoy. During the whole time we also recorded the song of the territory owner using a Sennheiser directional microphone (ME66/K6) connected to a Marantz solid state recorder PMD 660. Usually we could determine the location of the male during the whole STI, however, sometimes it was hidden from view and we could not correctly record head nods and fluffing behaviour. Therefore, we also noted when we knew the location of the bird but could not see it. After 20 min the playback was remotely muted and the decoy removed and the behaviour of the territory owner observed for another 10 min. After the STI we recorded the time the territory owner spent within 10 m of the decoy instead of 5 m. Behavioural observations were conducted blind to the treatment of the focal males and always by the same observer from at least 30 m distance. Song recordings were made by a second observer.

### Song analysis

Song was analysed using again Avisoft-SASLab Pro software, version 4.51. Recordings were visualized in spectrograms (settings: sample rate 22,050 Hz, FFT = 256 points, Hamming-Window, Overlap: 50%). We determined the number of songs by visual inspection and selected songs solely based on their recording quality (low background noise) for further sound analysis. Each song of black redstarts can be divided into three distinct parts (part A, B and C, see [24,26] and Figure 3 for more details) with a pause of varying duration between part A and B. We measured the duration of parts A, B, C, the total song and the duration of pauses between A and B. We counted the number of elements of part A and C (mean of max. 20 songs). We also determined the frequency bandwidth and the maximum frequency of part A, B and C using the automatic parameter measurement function (threshold -20 dB) in Avisoft (mean of max. 10 renditions of high-quality songs).

### Statistical analysis

Data analysis was done with R version 2.9.1 [55]. Behavioural data and measures of song structure were analysed with linear mixed models for the effects of treatment and day after implantation. We analysed the behaviour during and after the STI separately. To control for repeated measures we included bird ID as a random effect. After the STI we compared the time spent within 10 m of the decoy instead of 5 m as most males left the immediate surroundings of the decoy and went to higher singing posts. Also, because most males stopped feather fluffing after the decoy had been removed, we only analysed the number of head nods after the STI. When analysing the time within 5 m during the STI we included response latency as covariate in the models. For the analysis of treatment effects on the number of head nods and time spent feather fluffing we controlled for differences in the total amount of time we actually saw the bird. For treatment effects on song structural parameters we included the average duration of a song part as covariate in our models. Based on previous findings we assume that structural changes in the song in response to a simulated territorial intruder result in song of higher competitive value than song produced spontaneously [26].

In all cases we started with full models and removed interactions if they were above α > 0.1. Experimental factors were always retained in the models. Dependent variables were transformed if assumptions of normality and/or equality of variances were not met. Significance was accepted at α ≤ 0.05. Sample sizes may deviate from 20 males in total as not all males sang during the STI and depending on the quality of the recording.

## Competing interests

The authors declare that they have no competing interests.

## Authors’ contributions

BA co-conceived of the study, contributed to the design of the experiment and the field work, performed the hormone and statistical analysis, and co-drafted the manuscript. KGM contributed to the design of the experiment and the field work, co-conducted the song analysis, and co-drafted the manuscript. SaK contributed to the design of the experiment and the field work, co-conducted the song analysis, and co-drafted manuscript. SiK co-conceived of the study, contributed to the design of the experiment, co-conducted the song analysis and co-drafted the manuscript. WG co-conceived of the study, contributed to the design of the experiment and the field work, and co-drafted the manuscript. All authors approved the final manuscript.
